# The Central Role of Glucokinase in Glucose Homeostasis: A Perspective 50 Years After Demonstrating the Presence of the Enzyme in Islets of Langerhans

**DOI:** 10.3389/fphys.2019.00148

**Published:** 2019-03-06

**Authors:** Franz M. Matschinsky, David F. Wilson

**Affiliations:** Department of Biochemistry and Biophysics, Perelman School of Medicine, University of Pennsylvania, Philadelphia, PA, United States

**Keywords:** diabetes, metabolic regulation, glucose homeostasis, counter regulatory hormones, glucokinase

## Abstract

It is hypothesized that glucokinase (GCK) is the glucose sensor not only for regulation of insulin release by pancreatic β-cells, but also for the rest of the cells that contribute to glucose homeostasis in mammals. This includes other cells in endocrine pancreas (α- and δ-cells), adrenal gland, glucose sensitive neurons, entero-endocrine cells, and cells in the anterior pituitary. Glucose transport is by facilitated diffusion and is not rate limiting. Once inside, glucose is phosphorylated to glucose-6-phosphate by GCK in a reaction that is dependent on glucose throughout the physiological range of concentrations, is irreversible, and not product inhibited. High glycerol phosphate shuttle, pyruvate dehydrogenase, and pyruvate carboxylase activities, combined with low pentose-P shunt, lactate dehydrogenase, plasma membrane monocarboxylate transport, and glycogen synthase activities constrain glucose-6-phosphate to being metabolized through glycolysis. Under these conditions, glycolysis produces mostly pyruvate and little lactate. Pyruvate either enters the citric acid cycle through pyruvate dehydrogenase or is carboxylated by pyruvate carboxylase. Reducing equivalents from glycolysis enter oxidative phosphorylation through both the glycerol phosphate shuttle and citric acid cycle. Raising glucose concentration increases intramitochondrial [NADH]/[NAD^+^] and thereby the energy state ([ATP]/[ADP][Pi]), decreasing [Mg^2+^ADP] and [AMP]. [Mg^2+^ADP] acts through control of K_ATP_ channel conductance, whereas [AMP] acts through regulation of AMP-dependent protein kinase. Specific roles of different cell types are determined by the diverse molecular mechanisms used to couple energy state to cell specific responses. Having a common glucose sensor couples complementary regulatory mechanisms into a tightly regulated and stable glucose homeostatic network.

In the spirit of Claude Bernard’s dictum: “Particular facts are never scientific, only generalization can establish science”.

## Introduction

**Figure 1:**
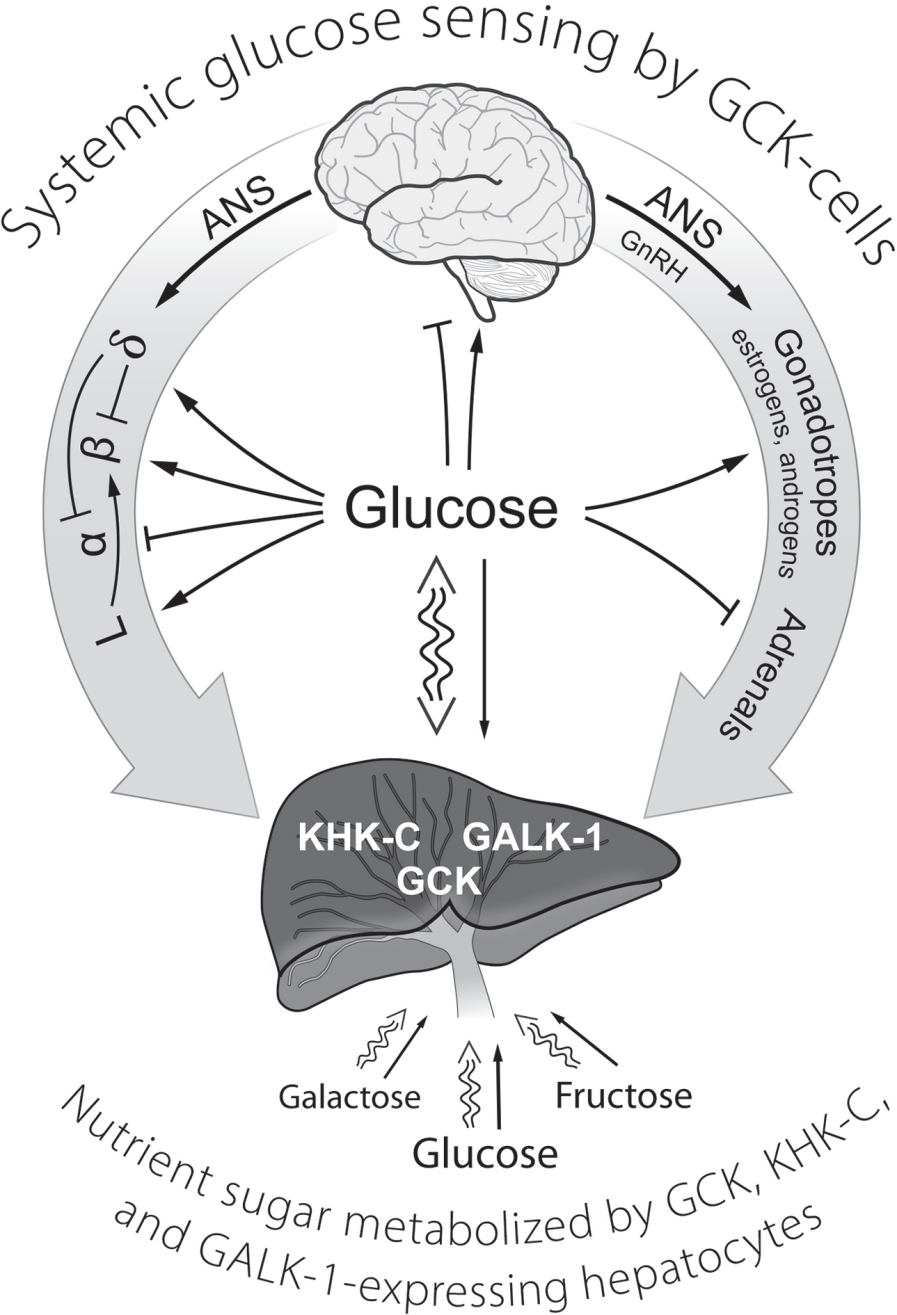
A schematic representation of GCK containing cells in sensing systemic glucose and regulating glucose homeostasis. Systemic glucose concentration is sensed by cells through its metabolism by GCK and the rate of production of G-6-P on cellular energy state. Cell-specific mechanisms then translate alterations in energy state into responses (hormone release, neural activity) that either augment or inhibit glucose consumption and/or production of glucose as appropriate to maintenance of homeostasis. The central role of the liver in metabolizing dietary sugars (glucose, galactose, and fructose), nearly quantitative removal of the galactose and fructose, is noted. ANS, autonomic nervous system; GCK, glucokinase; GALK-1, galactokinase-1; KHK-C, ketohexokinase-C (i.e., fructokinase); GLP-1-producing enteroendocrine L-cells; GnRH, gonadotropin-releasing hormone; α, β, δ, the alpha, beta, and delta cells of pancreatic islets.

Our hypothesis is that glucokinase (GCK) is the primary glucose sensor in mammals and the sensor responsible for regulation of glucose homeostasis as schematically represented in Figure 1. That the signals for decreasing blood glucose (insulin) and for increasing blood glucose (glucagon, epinephrine, and other counter regulatory hormones and transmitters) depend on a common glucose sensor greatly facilitates integration into a stable regulatory system. In support of our hypothesis, we will systematically develop its scientific basis. The steps involved are: (1) evidence leading to recognition of the problem (Introduction). (2a) Establishing a positive correlation between the presence (and activity) of GCK and glucose response by cells that contribute significantly to regulation of glucose homeostasis and (2b) establishing that cells which do not respond directly to changes in blood glucose have little or no GCK activity or role in glucose homeostasis. (2c) Evaluating the special metabolic role of the liver which contains most of the body’s GCK activity. (3) Establishing causality, e.g., that alterations in GCK activity directly and in predictable ways affect glucose homeostasis. (4) Evaluate evidence that glucose homeostasis utilizes glucose sensors other than GCK. We have included our definitions of receptor and metabolic metabolite sensing in order to avoid the ambiguity that might otherwise arise.

## Background and Origin of the GCK Glucose Sensor Paradigm

A critical role of d-glucose in regulating blood sugar levels was first recognized in 1927 by Grafe and Meythaler (1927a,b) in seldom cited reports. They were thus first to propose a primary messenger function for blood glucose as indicated by the title of their paper: “Traubenzucker als Hormone” (“grape sugar as a hormone”). The authors had arrived at this concept because relatively small amounts of d-glucose infused into the pancreatico-duodenal artery of dogs caused lowering of systemic blood sugar. Mechanistic explanations, as well as full understanding and appreciation for this seminal observation progressed, but rather slowly. A critical milestone was reached when Grodsky and colleagues (Grodsky et al., 1963, 1967) reported that d-glucose stimulates insulin secretion from the isolated perfused rat pancreas while Coore and Randle (1964a,b), using pieces of rabbit pancreas, reported that d-glucose stimulates insulin secretion and that metabolism of glucose was apparently required for insulin release. In 1968, Matschinsky and Ellerman (1968) reported that glucokinase (GCK, hexokinase 4, EC.2.7.2.1), known since 1963 to play a critical role in hepatic glucose metabolism (Salas et al., 1963; Sharma et al., 1963), is present in insulin producing islets of Langerhans of obese mice (OB/OB C57Bl 6 J mice). This laid a firm foundation for understanding the metabolic basis of glucose sensing. Matschinsky and Ellerman (1968) also showed that intra and extracellular glucose of islet tissue equalized rapidly, indicating glucose transport was not rate limiting for metabolic glucose sensing. The presence of glucose-6-phosphatase activity comparable to that of GCK, despite low glycogen content not affected by increasing glucose, was also observed. Raising blood glucose of mice was observed to profoundly change the metabolite and cofactor profiles in the pancreatic islets. The similarities to liver were recognized while noting crucial differences, such as the low glycogen level and lack of its response to glucose. Possible mechanisms for how glucose metabolism by GCK and altered metabolite profiles could trigger insulin secretion were considered but possible mechanisms for stimulus secretion coupling were only speculative at this point (Matschinsky et al., 1968; Matschinsky and Ellerman, 1968; Ashcroft et al., 1973). Ashcroft and Randle (1968) found that glucose-6-phosphate catabolism by glucose-6-phosphatase and by the pentose-phosphate-shunt (Ashcroft et al., 1972) were barely augmented by raising glucose, indicating that glycolysis was the primary route of glucose metabolism in islet cells. It was nearly 20 years before Bedoya et al. (1985, 1986b) were able to demonstrate activity of GCK in human pancreatic islet tissue comparable to that found in mice and rats, measurements made possible by development of highly sensitive and specific radiometric methods that eliminated interference by other hexokinases. In the meantime, Dean and Matthews (1970) and Meissner and Schmelz (1974) reported that glucose lowered the β-cell membrane potential and induced electrical activity, a critical step toward understanding of how glucokinase activity could be coupled to insulin release. This was followed by the seminal observation by Ashcroft and coworkers (Ashcroft et al., 1984; Ashcroft, 2005) that glucose induced closure of a K^+^_ATP_ channel in pancreatic β-cells, strongly suggesting that cellular energy state ([ATP]/[ADP][Pi]) is responsible for coupling glucose stimulus to insulin secretion. Through the efforts of many laboratories, it was established that calcium and cyclic AMP served as second messengers in stimulus secretion coupling of β-cells (Malaisse et al., 1967; Sussman and Vaughan, 1967; Turtle and Kipnis, 1967; Charles et al., 1973; Zawalich et al., 1975; Wollheim and Sharp, 1981; Liang et al., 1996). This extensive knowledge base allowed Matschinsky and coworkers (Matschinsky and Ellerman, 1968; Trus et al., 1980, 1981; Meglasson et al., 1983; Meglasson and Matschinsky, 1986; Matschinsky, 1990; Ghosh et al., 1991; Matschinsky et al., 1993; Sweet and Matschinsky, 1995; Sweet et al., 1996) to conceptualize a metabolic glucose sensing system involving GCK activity, oxidative phosphorylation, K^+^_ATP_ channels, and voltage gated calcium channels. Advances in human genetic analysis soon established linkage between the GCK gene and certain forms of MODY (Maturity Onset Diabetes in the Young) by Froguel et al. (1993) and Hattersley et al. (1992), and of HI (Hyperinsulinism) by Glaser et al. (1998), respectively. This linkage demonstrated the medical relevance of the GCK glucose sensor concept. Soon thereafter mouse models were established, facilitating detailed experimental characterization of GCK and its links to diabetes mellitus and hypoglycemia (Epstein et al., 1992; Grupe et al., 1995; Terauchi et al., 1995; Niswender et al., 1997). The 2003 discovery of allosteric GK activator drugs (so called GKAs) by Grimsby et al. (2003) at Hoffman La Roche was a crucial achievement. It provided strong pharmacological support for the GK glucose sensor paradigm as well as a new experimental tool and hope for a new treatment of Diabetes Mellitus. The potential of GKAs has since been vigorously explored both by industry and academia (Matschinsky, 2009, 2013). A minimal computational model for GCK based β-cell glucose sensing has been recently developed that emphasizes the essential roles of 5’-AMP and MgADP as metabolic coupling factors in glucose stimulation of insulin release (Wilson et al., 2017, 2018).

During development of the GCK glucose sensor hypothesis, primary focus was on insulin producing β-cells for both conceptual and practical reasons; conceptual, because insulin is arguably the dominant regulator of systemic glucose metabolism and practical, because β-cells are abundant and experimentally accessible. Glucagon, discovered in 1923 by Kimball and Murlin (1923), was recognized early on as another important player in glucose homeostasis, establishing that control of glucose homeostasis required two hormones (Grodsky et al., 1967; Unger and Orci, 1975; Gromada et al., 2007; Pullen et al., 2011; Unger and Cherrington, 2012). In contrast to the situation with insulin, increased glucagon increases glucose production and increase in blood glucose suppresses release of glucagon. Many investigators share the view that glucose suppression of glucagon is indirect, mediated through paracrine action of insulin, somatostatin or other factors released by neighboring cells (e.g., Zn ions). There are, however, several lines of evidence, including the clearly divergent glucose dependency curves of glucagon suppression and insulin secretion and glucose induced suppression of glucagon release from islets from diabetic animals (Matschinsky et al., 1976), which indicate glucose directly affects α-cells. This suggested that GCK might also serve as glucose sensor in α-cells (Bedoya et al., 1985, 1986a,b, 1987; Heimberg et al., 1996). Basco et al. (2018) have seemingly settled this question by demonstrating that knocking out GCK specifically in mouse α-cells blocks glucose-dependent suppression of glucagon and results in a hyperglycemic state.

## The GCK Glucose Sensor Paradigm in a Nutshell

Evidence supporting the hypothesis that GCK is responsible for coupling glucose concentration to insulin release of β-cells, and that it acts by altering cellular energy state, is now extensive (see Matschinsky and Ellerman, 1968; Trus et al., 1980; Meglasson et al., 1983; Ghosh et al., 1991; Liang et al., 1992, 1996; Matschinsky et al., 1993; Sweet and Matschinsky, 1995; Terauchi et al., 1995; Ferre et al., 1996; Sweet et al., 1996; Schuit et al., 1997; Detimary et al., 1998; Pino et al., 2007; Osbak et al., 2009; Sayed et al., 2009; Doliba et al., 2012; Prentki et al., 2013; Nicholls, 2016; Morishita et al., 2017; Wilson et al., 2017, 2018; Affourtit et al., 2018; Zhu et al., 2018). A computational model has been developed that is consistent with regulation of oxidative phosphorylation *in vivo* (Ox-Phos model) and its role in metabolic homeostasis (Wilson and Vinogradov, 2014, 2015; Wilson, 2015a,b, 2016, 2017a,b). This Ox-Phos model has been shown to predict behavior consistent with experimental measurement over a wide range of conditions, including those during the rest-to-work (Wilson, 2015b) and work-to-rest (Wilson, 2016) transitions in skeletal muscle. A model for glucose sensing was then constructed by adding a computational model for GCK and glycolysis in pancreatic β-cells, including coupling to oxidative phosphorylation through the glycerol phosphate shuttle and pyruvate dehydrogenase, to the Ox-Phos model (Wilson et al., 2017, 2018). The combined models quantify how increasing glucose concentration, through increased synthesis of glucose-6-phosphate by GCK, increases the energy state and decreases [Mg^2+^ADP] and [AMP]. Glucose is transported into the cells by facilitated transport with sufficiently high capacity (Matschinsky et al., 1968, 1976; Pullen et al., 2011) that there is rapid equalization between extracellular and intracellular glucose concentrations. Glucokinases from different species have very different dependency on glucose concentration (half saturation 1.5–12 mM, n = 1.4–1.7) than do the other three mammalian hexokinases (half saturation 0.05–0.2 mM, n = 1) and unlike the latter is unaffected by physiological concentrations of product (Cárdenas, 2004). The corresponding values of these kinetic constants reported for human GCK are about 8.0 mM and n = 1.7, respectively. As a result, increasing blood glucose increases production of G-6-P (and practically all downstream glycolytic metabolites) by GCK. It is primarily the increase in F-6-P that activates phosphofructo-6-kinase, and thereby glycolysis, while F-2,6-P2, which regulates this enzyme in hepatocytes, changes little if at all when β-cell glucose is increased (Truehart-Burch et al., 1985). β-Cells have high glycerol phosphate shuttle activity, low lactate dehydrogenase activity, low plasma membrane monocarboxylate transporter, limited pentose phosphate shunt and G-6-P phosphatase activity, and low capacity to store glucose as glycogen. The high glycerol phosphate shuttle and low lactate dehydrogenase activities constrain glycolysis to primarily producing pyruvate. Low monocarboxylate transport both minimizes pyruvate loss through transport to the extracellular medium and prevents increased blood lactate levels, as during exercise, from interfering with glucose sensing (Otonkoski et al., 2007; Pullen et al., 2011; Prentki et al., 2013). The pyruvate produced from glucose is primarily oxidized through pyruvate dehydrogenase (PDH) or carboxylated by pyruvate carboxylase. Increasing glucose concentration increases glycolysis and thereby input of reducing equivalents both into the citric acid cycle through PDH and directly into the respiratory chain through the glycerol phosphate shuttle. [*Note: if the glycerol phosphate shuttle is blocked the glutamate-malate shuttle can take over, albeit with some loss of control.*] The influx of reducing equivalents is greater than what is needed for ATP synthesis, as measured by oxygen consumption, and is known to be a function of the glucose stimulus. The influx of reducing equivalents causes reduction of the intramitochondrial NAD pool, increase in energy state ([ATP]/[ADP][Pi]), not surprisingly manifest primarily in decreased [Mg^2+^ADP] and [AMP] with little change in [ATP]. Decrease in [Mg^2+^ADP] inhibits ATP-dependent K^+^ ion channels, depolarizing the plasma membrane, increasing intracellular Ca^2+^, and releasing insulin. Lowering of 5’-AMP removes the brake that AMP-kinase appears to impose on basal insulin release (Rutter et al., 2003). It is important to note that the glucose induced increase in oxygen consumption is not due to increase in energy state *per se*, but to energy consuming reactions activated by the increase in energy state (see Wilson et al., 2017, 2018). The increase is probably due to increased ion transport secondary to altered K^+^ channel conductance as augmented by increased biosynthetic activity and possibly activation of energy consuming substrate cycles (Prentki et al., 2013). The increase in ATP use is, however, variable among species and the mechanisms that couple glucose-dependent increase in energy state to increased energy consumption remain to be characterized and quantified.

**Figure 2:**
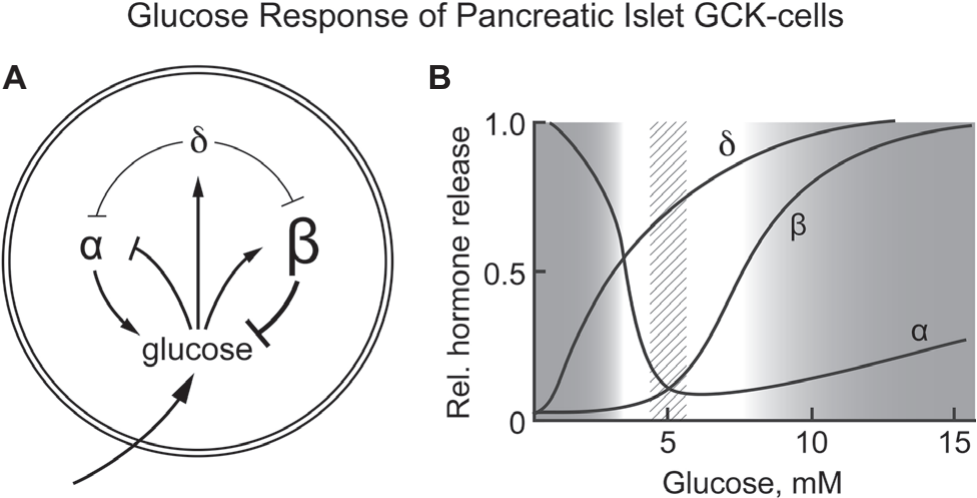
**(A,B)** Response of pancreatic α, β, and δ-cells to systemic glucose concentration and the relationship of hormone release by each cell type as a function of blood glucose levels. Schematically represents the role of each cell type in controlling blood glucose concentration. Increasing glucose suppresses glucagon release by α-cells while increasing insulin release by β-cells. It also increases release of somatostatin from δ-cells, a paracrine signal that suppresses hormone release by both α- and β-cells. **(B)** schematically shows the relative amount of hormone released at each glucose concentration. A dotted vertical bar is shown at 5 ± 0.5 mM, the set point for glucose homeostasis. **(B)** is based on graphic depictions (Matschinsky et al., 1976; Gylfe, 2016; Rorsman and Huising, 2018).

Glucose homeostasis involves a complex regulatory system that includes, in addition to pancreatic β-cells, a number of other cells/tissues, notably α- and δ-cells of pancreatic islets, entero-endocrine cells, the adrenal glands, glucose sensing neurons in the CNS, the pituitary gland, and the liver. Our hypothesisis is that GCK is responsible for sensing blood glucose not only by pancreatic β-cells but also by the other cells of the integrated system (mostly counter regulatory) responsible for regulating blood glucose. Corollaries to this hypothesis are: (1) the metabolism that couples GCK activity to altered energy state is similar for all glucose sensing cells, and (2) specific responses appropriate to the role of each cell/tissue in glucose homeostasis are determined by differences in how the energy state is coupled to cellular response and nature of the cellular response (cell specific hormone release, neural activity, etc). Figure 2 schematically presents pancreatic responses to glucose and how the glucose induced hormonal responses provide a homeostatic set point for blood glucose.

## Definition of Metabolite Sensors as Used in This Paper

It is important to define metabolite sensing and sensors. Not everyone uses the same definitions, and giving our definitions at the outset may avoid misunderstanding due to semantic differences. In our view the term “metabolite sensor” applies only to the entity that directly interacts with the metabolite and converts its concentration into signal (s) that are chemically and/or physically different from the original metabolite. All entities that respond, not to the metabolite itself, but to signal(s) generated by the sensor are referred to by terms appropriate to their function: part of the downstream signaling pathway, signal response elements, etc. Evolution has developed two basic mechanisms for measuring and responding to (sensing) levels of critical metabolites in the cellular environment:

### Receptor-Based Metabolite Sensors

Receptor based metabolite sensing detects metabolite levels using elements, typically proteins, which selectively bind the metabolite. Receptors respond to binding of the metabolite (agonist) with a structural change that activates its downstream signaling pathway. In general, receptor mediated metabolite sensing does not require or involve chemical alteration of the metabolite to activate downstream signaling. Response to metabolite binding may include altered membrane permeability, protein kinase or phosphatase activity, lipase activity, gene expression etc and these dictate the cellular response. Receptor mediated sensing is widely used in neurotransmission, hormonal regulation, etc. (amino acid, fatty acid, and cholesterol receptors as well as leucine stimulation of mTor are good examples (Efeyan et al., 2015).

### Metabolism-Based Metabolite Sensors

Metabolism mediated sensing consists of an enzyme that chemically alters the messenger/signal metabolite, typically in an irreversible reaction. It is the rate of production of reaction product(s) that provides a measure of the sensed metabolite. Downstream signal intensity is dependent not on the metabolite itself but on the metabolic products. The downstream signaling pathways, however, may in the end be the same as for receptor mediated sensing and include alterations in membrane permeability, protein kinases, protein phosphatases, lipases, gene expression, etc. In addition, the reaction products themselves may activate or inhibit metabolism not directly related to that of the original metabolite. To be classed as a metabolic sensor, the enzyme activity should play a significant role in regulating metabolism other than its own. Important examples of metabolites for which metabolic sensing has profound regulatory contributions are glucose (Matschinsky, 1990, 2009, 2013; Matschinsky and Ellerman, 1968; Matschinsky et al., 1968, 1993, 1976) and oxygen (Wilson et al., 1994; Wilson 2017b). Using these definitions, metabolic sensors clearly include enzymes where metabolite induced activity is coupled to, and modulates, levels of downstream regulatory factors (metabolic coupling factors or second messengers). These lower tier messengers then modulate enzyme activities, gene expression, or ion permeability in order to regulate metabolism of the stimulant itself and/or other metabolites including amino acids or fatty acids. This definition of metabolic sensors allows separation of metabolic sensors from enzymes in general, but necessarily leaves a “gray area” which includes some enzymes for which classification will be ambiguous. The role of GCK in liver metabolism is such a case and this will be discussed in some detail (see below).

In the present paper, focus is on glucose homeostasis and glucose sensing in mammals will be used as our model for metabolism mediated sensors. Mammalian diets contain many different sugars, most prominently glucose, fructose, and galactose. Evolution has resulted in carbohydrate metabolism being based on glucose. This may well be because d-glucose is the most abundant low molecular carbohydrate building block in nature. Fructose and galactose, nutritionally important hexoses that enter portal blood from the diet, are nearly quantitatively cleared by fructokinase-C (Hayward and Bonstron, 1998) and galactokinase-1 (Bergsma et al., 1996), respectively, in one passage through the liver. This decreases their concentrations in systemic blood to near zero. Glucose, in contrast, is maintained near 5 mM in blood by an elaborate regulatory system and disturbances of this regulatory system give rise to a wide range of pathologies (hypoglycemia or diabetes mellitus). Endocrine pancreatic cells, through control of insulin and glucagon levels, play a preeminent role in regulation of blood glucose and are the most studied factors of glucose homeostasis. Solid evidence has been obtained that in pancreatic β-cells, which are responsible for release of insulin, the glucose level is sensed by GCK as discussed above. Evidence that GCK also serves as sensor for glucose suppression of glucagon secretion from α-cells has been accumulating slowly but the view that glucose acts directly on these cells *via* GCK is becoming widely held (Bedoya et al., 1986a,b; Heimberg et al., 1996; Gromada et al., 2007; Le Marchand and Piston, 2012; Gylfe, 2016; Basco et al., 2018). The metabolic consequences of varying glucose concentration, and thereby GCK activity, on β-cell metabolism are quite well understood. A model is available that quantifies how glucokinase activity, coupled to oxidative phosphorylation, regulates insulin release (Wilson et al., 2017, 2018). Our current understanding of glucose sensing by glucokinase in β-cells and metabolism which couples GCK activity to insulin release has been outlined above. It is important to note that increase in energy state with increase in glucose concentration, through near equilibrium of adenylate kinase, also markedly decreases [AMP]. As a result of equilibration of adenylate kinase ([AMP] = K [ADP]^2^/[ATP]), [AMP] decreases approximately as [ADP]^2^, making AMP a very sensitive measure of energy state. Decrease in [AMP] has many consequences, a prominent example being to suppress the activity of AMP-dependent protein kinase (AMPK). AMPK is an important regulator of many different cellular functions, in particular energy metabolism (Rutter et al., 2003; Hardie et al., 2012; Sun et al., 2015; Rajamohan et al., 2016; Hardie and Lin, 2017). It was recently reported that the glycolytic enzyme aldolase binds to, and activates, AMPK and that binding of fructose-1,6-bisphosphate (F-1,2-P2) to aldolase results in dissociation of the aldolase-AMPK complex, deactivating the kinase (Hardie and Lin, 2017). Increasing glucose concentration greatly increases F-1,2-P2 of β-cells (Matschinsky and Ellerman, 1968; Trus et al., 1981) raising the possibility this is an additional mechanism for AMPK regulation (see Figure 1 in Wilson et al., 2018). In β-cells, AMPK activity is reported to suppress insulin release (Rutter et al., 2003). Glucose concentration-dependent changes in [AMP], and perhaps F-1,2-P2, provide “second tier” regulation of hormonal release/neural output by sensory cells involved in regulating glucose levels.

## Correlation Between the Presence of GCK and Glucose Response by Cells Contributing to Glucose Homeostasis

Many different, functionally specialized glucose sensing cell types have evolved to assure blood glucose is maintained within a narrow, physiologically safe range of 4–8 mM. These cells complement each other in providing continuous measure and adjustment of the concentration of glucose in blood. We hypothesize that GCK is the primary glucose sensor utilized for maintaining glucose homeostasis, i.e., GCK is responsible for sensing blood glucose not only by β-cells but also by other cells involved in regulating blood glucose. In the case of α-cells, a recent and striking advance was made by specifically deleting GCK from the α-cells in mice, the deletion resulting in substantial loss of glucose induced suppression of glucagon release (Basco et al., 2018). The resulting hyperglucagonemia causes overactive glucagonogenesis, consistent with metabolism of glucose by GCK having a central role in α-cell function in glucose homeostasis. The authors conclude that glucose sensing is by GCK and that downstream signaling in α-cells is similar to that of β-cells: increased glucose increases the [ATP]/[ADP] ratio leading to closure of ATP-regulated K^+^ channel and membrane depolarization. In α-cells, however, depolarization leads to voltage-dependent inactivation of voltage-gated Na^+^ channels involved in action potential firing. Diminution of action potential height decreases activation of the P/Q Ca^2+^ channels that mediate Ca^2+^ entry, and this decreases glucagon release. Note that Le Marchand and Piston (2012), have provided evidence that glucose induced suppression of glucagon secretion does not involve decreased intracellular [Ca^2+^].

Pancreatic δ-cells respond to increased glucose by releasing somatostatin which acts locally as a paracrine inhibitor of insulin and glucagon release rather than a systemic hormone (Boden et al., 1981, Gylfe, 2016; Rorsman and Huising, 2018). Glucose sensing by δ-cells most likely involves GCK for sensing as indicated by substantial expression of the gene as measured by single cell RNA-seq. It should be noted that pancreatic α-, β-, and δ-cells have a common progenitor cell (Sosa-Pineda et al., 1997; Gradwohl et al., 2000; Chung and Levine, 2010) which could help explain the obvious similarities in their glucose sensing pathways. δ-cells also respond directly to a physiological amino acid mixture independently of glucose, perhaps the consequence of membrane depolarization caused by sodium co-transport being sufficient to reach the threshold for secretory response (Boden et al., 1981; Rorsman and Huising, 2018).

Similar GCK-dependent sensing paths have been proposed for glucose excited (GE) and glucose inhibited (GI) neurons of the CNS. Dunn-Meynell et al. (2002) reported that GCK mRNA is expressed in norepinephrine neurons of locus ceruleus, and in hypothalamic neuropeptide Y, pro-opiomelanocortin, and γ-aminobutyric acid neurons. Moreover, in GE neurons intracellular Ca^2+^ oscillations were inhibited and in GI neurons Ca^2+^ oscillations were stimulated by four selective GCK inhibitors. Lamy et al. (2014) studied GLUT2-expressing excitatory neurons (GE) in the nucleus of tractus solitarius of the vagus nerve. These neurons form a distinct population of hypoglycemia-activated neurons. The authors concluded that glucose is sensed through GCK because hypoglycemia decreases energy state and increases [AMP]. Increased [AMP] then activates AMP-dependent protein kinase and this suppresses a K^+^ leak current, hyperpolarizes the cells, and increases afferent electrical activity. Similar results have been presented for GI neurons of the hypothalamic arcuate nucleus (Burdakov et al., 2005; Routh, 2010; Hussain et al., 2015). There are a large number of different types of neurons for which glucose sensitivity has been reported (Levin et al., 2004; Jordan et al., 2010; Thorens, 2012; Lamy et al., 2014; Steinbusch et al., 2015, 2016) and in most of these neurons glucose sensing appears to be through GCK, although orexin neurons of the hippocampus are apparently an exception (see section on alternate glucose sensors).

GCK has also been implicated in glucose sensing in the anterior pituitary gland of rat and monkey (Zelent et al., 2006; Sorenson et al., 2007). GCK activity was measured spectrophotometrically in whole pituitary extracts and identified as a generalized cytoplasmic staining co-localized in cells with follicle-stimulating hormone (FSH) or luteinizing hormone (LH). In addition to gonadotropes, GCK was observed in a minor subpopulation of corticotropes and thyrotropes (Sorenson et al., 2007). It is puzzling, however, that in gonadotropes of the cynomolgus monkey GCK appeared to be clearly confined to cytosolic substructures, tentatively identified as Golgi complex (Sorenson et al., 2007). Pulsatile stimulation of the gonadotropes FSH and LH is mediated by GNRH released from GNRH neurons of the preoptic nucleus, which in turn are regulated by hypothalamic Kisspeptin neurons. GNRH receptor activation results in phospholipase C activation, IP3 mediated ER-calcium release causing secretion of the hormones (Stojilkovic et al., 2005). It remains to be tested whether, and if so how, glucose might influence these cells as they control puberty and/or reproduction. Nutrient supply profoundly influences reproductive biology, and GCK-dependent glucose regulation at the level of the pituitary might play a hitherto not considered role. Note that in the case of gonadotropes, glucose sensing might influence the release of FSH and/or LH which would impact glucose homeostasis directly *via* sex steroids or indirectly through increased nutrient consumption due to the pubertal growth spurt or pregnancy.

A case can also be made that GCK is the glucose sensor for cells in the adrenal gland which secrete epinephrine and corticosteroids to counteract hypoglycemia. Three lines of evidence support this contention. A rather mild form of hypoglycemia, observed clinically, later found to be caused by an activating mutation of GCK (M197 T?), was initially diagnosed as adrenal insufficiency. The patient was treated for 12 years with a maintenance dose of 10 mg cortisol to ameliorate hypoglycemia symptomatology (Morishita et al., 2017). After reevaluation of the original diagnosis, steroid treatment was discontinued because hypertension had developed and adrenal insufficiency was discounted. It is noteworthy that this particular GCK mutation increases activity of the enzyme only moderately as indicated by a thorough kinetic analysis (Sayed et al., 2009). It is therefore not unreasonable to hypothesize the clinical presentation resulted from complex additive effects of GCK activation that involved altered glucose sensing by both pancreatic α- and β-cells as well as cells of the adrenal gland. This interpretation is strengthened by a recent demonstration of GCK mRNA in mouse adrenal glands (see Supplement figure in Steinbusch et al., 2016) and immunohistochemical data indicating GCK is present in cells of the human adrenal gland (online Human Atlas showing histochemical staining of GCK).

These examples illustrate that each cell type having a role related to blood glucose levels expresses GCK and there is evidence that glucose is sensed through the activity of GCK. Our hypothesis predicts that all utilize similar metabolism to couple GCK activity to energy state and their metabolic response to changes in glucose concentration closely approximate those in pancreatic α- and β-cells and in glucose sensing neurons. The mechanism and metabolism related to glucose sensing is similar for all of the cells, while differences in coupling of energy state to membrane permeability, specific hormone and/or neurotransmitter released determining outcome and role in glucose/energy metabolism regulation. Examples of how similar changes in energy state elicit different cell specific responses have been presented for β-cells, α-cells, and glucose sensing neurons.

It is also important that GCK is absent or very low in cells and tissues that do not serve as glucose sensors as defined. These cells express hexokinase instead of GCK and the higher affinity for glucose (0.05–0.2 vs 1.5–12 mM, (Cárdenas, 2004)) results in cellular metabolism being insensitive to changes in glucose concentration in the physiological ranges found in different species. Zelent et al. (2006), for example, measured expression of GCKmRNA in islets of Langerhans (pancreas), pituitary, adrenal gland, whole brain, acinar pancreas, liver, skeletal muscle, adipose tissue, lung, kidney, and spleen. Of these, GCK mRNA was expressed in substantial amounts in the islets of Langerhans, pituitary, and liver with only trace amounts in the other tissues. Even more telling is that in brain, GCK mRNA is expressed in only a very small fraction of neurons and then only in neurons that respond to changes in physiological glucose concentrations (see Hussain et al., 2015; De Backer et al., 2016).

## Dual Control of GCK Expression and its Implications for Understanding Glucose Homeostasis

In this paper, focus is on glucose homeostasis and glucose sensing in mammals. As noted earlier, mammalian diets contain many different sugars, most prominently glucose, fructose, and galactose but mammalian internal carbohydrate metabolism is based on glucose. Fructose and galactose are nearly quantitatively cleared from the portal blood in one passage through the liver, decreasing their concentrations to near zero. Glucose, in contrast to the other two hexoses, is maintained near 5 mM in the blood. Displacement from this level, typically associated with altered insulin production or efficacy, gives rise to a wide range of pathologies. Endocrine pancreatic cells, through control of insulin and glucagon levels, play a dominant role in regulation of blood glucose and are the most studied and best understood factors of glucose homeostasis. The insulin/glucagon ratio (I/G ratio) determines the relative rates of hepatic glycogen synthesis, glycogenolysis, and gluconeogenesis by regulating the activity and content of enzymes in the opposing pathways, i.e., glucokinase vs glucose-6-phosphatase and glycogen synthase vs phosphorylase.

Control of GCK expression is now well established to involve a single gene with two organ specific promoters (Iynedjian, 2009), A constitutively active upstream neuroendocrine promoter (NE-GCK-promoter) controls glucokinase expression in endocrine pancreas, brain, pituitary, adrenal and entero-endocrine cells and a separate, insulin-dependent downstream promoter controls expression in liver (HEP-GCK-promoter). Differential splicing results in hepatic and neuro-endocrine GCK isoforms that are functionally indistinguishable. It is noteworthy, however, that there are differences in amino acid sequence at the N-terminal ends of neuroendocrine and hepatic forms of GCK, but as yet no functional relevance has been attributed to this difference. Bedoya and coworkers had discovered in 1986 (Bedoya et al., 1986a,b) that GCK expression was differentially regulated in liver and endocrine pancreas when characterizing the effects of a transplantable insulinoma on GCK expression in liver and islets of Langerhans in NEDH rats (New England Diakonis Hospital rats). The authors proposed that glucose directly affects GCK levels in β-cells (chronic exposure) and thereby modulates insulin synthesis, secretion and β-cell replication, predictions that have since been shown to be correct (Iynedjian, 2009). In general, NE-GCK-promoter controlled expression of GCK in glucose sensing cells is largely constitutive but the levels can be increased by glucose through post translational stabilization of the enzyme, decreasing turnover. In contrast, HEP-GCK-promoter controlled expression of GCK in liver is insulin dependent with no apparent impact of a wide range glucose levels (1–25 mM).

## The Special Role of Liver in Glucose Homostasis

**Figure 3:**
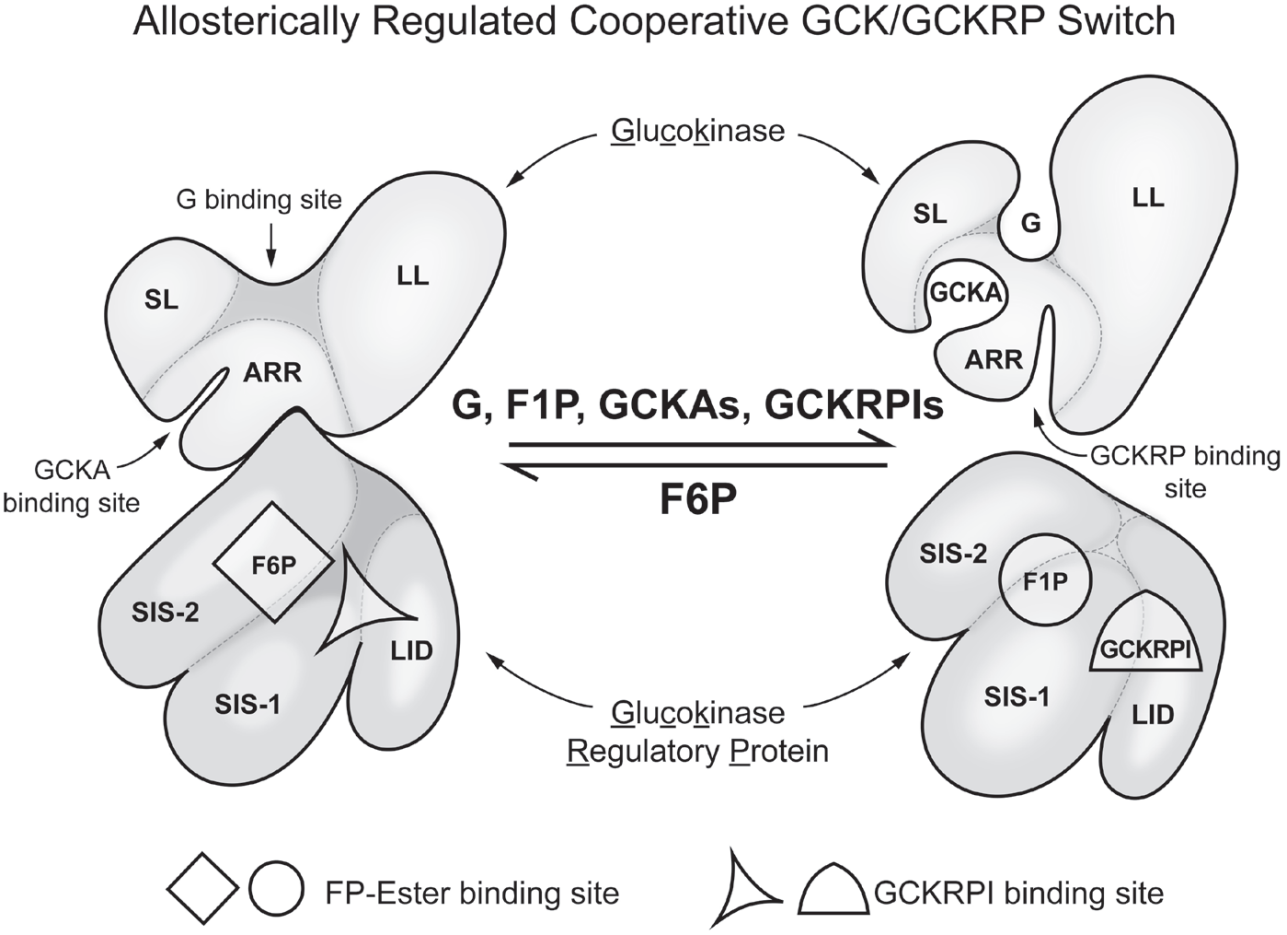
Regulation of glucokinse activity in liver by liver specific GCK regulatory protein (GCKRP). In hepatocytes, in contrast to glucose sensing cells in regulation of glucose homeostasis, there is a specific inhibitory protein that regulates GCK activity. The figure schematically represents the association of GCK and GCKRP and the metabolic factors that affect that interaction. This can be considered a “switch” that turns enzyme activity on when blood glucose in elevated and off when it is low relative to normal (for background and details, see Zelent et al. (2014). SL, small lobe; LL, large lobe; ARR, allosteric regulatory region; SIS-1, sugar isomerase; SIS-2, sugar isomerase-2; LID, lid.

Liver has many functions, both metabolic and regulatory, and these regulatory functions include release of hormones involved in regulating general nutrient status, such as FGF21 (Fisher and Maratos-Flier, 2016). The nutrient responses of the liver are not specific to glucose, however, and have only secondary effects on glucose homeostasis. Our focus is on glucose homeostasis and GCK. In contrast to neurons and endocrine tissues, in liver GCK has a major metabolic role, removing glucose from the blood when the levels are above normal. The removed glucose is largely stored as glycogen or used for fat synthesis. This not only helps to prevent postprandial hyperglycemia but also assures adequate liver glycogen stores to stabilize blood glucose levels between meals (Agius, 2016). Additional levels of regulation of GCK present in liver that are specific to its role in glucose homeostasis include: (1) it is inhibited by a liver specific protein, GCKRP, and (2) expression of the GCK gene is entirely dependent on insulin. GCKRP is a 65 kDa monomeric protein expressed in hepatocytes in three- to fourfold excess of GCK (mol/mol) and localized almost exclusively in hepatic cell nuclei. Human GCKRP inhibits human GCK in a manner competitive with glucose as measured *in vitro* when all three components are at physiological concentrations (de la Iglesia et al., 2000; van Schaftingen and Veiga da Cunha, 2004; Zelent et al., 2014; Agius, 2016). Figure 3 schematically shows some of the factors that affect interaction of GCK with GCKRP and contribute to regulation of GCK activity in hepatocytes. This inhibition is increased by fructose-6-phosphate (F-6-P) and reversed by fructose-1-phosphate (F-1-P), the product of fructokinase C. During fasting GCKRP binds GCK, inactivating it, and the complex is sequestered in the cell nucleus. It can be released by glucose or F-1-P following feeding, depending on the nature of consumed carbohydrate. Dissociation of GCK/GCKRP complex by glucose results when glucose binds to the substrate site of GCK but this effect of glucose does not require MgATP; i.e., glucose acts as a first messenger, inducing a structural change of GCK causing the dissociation of the complex. F-1-P, in contrast, dissociates the complex by binding to GCKRP at a specific sugar-phosphate binding site where it is competitive with F-6-P, which stabilizes the complex. Details of the molecular processes underlying nuclear sequestration and release of the proteins are complex and remain to be elucidated. Highly effective drugs (called GCKRP inhibitors or GCK/GCKRP disruptors) have been developed that dissociate the GCK/GCKRP complex and thereby stimulate hepatic glucose phosphorylation and this lowers blood sugar in diabetic animals (Ashton et al., 2014). It remains to be seen whether using these agents proves clinically useful in treatment of hyperglycemia syndromes in T2DM and T1DM.

The preceding paragraph illustrates that carbohydrate metabolism of liver occupies a unique position in the intricate control system that maintains glucose homeostasis in man. This role is clearly distinct from those of the various cells and organs comprising the endocrine and neuronal network that regulates blood glucose as characterized in earlier paragraphs. First, it must be appreciated that the hepatic involvement in regulating carbohydrate metabolism is only one of many functions liver plays in maintaining general body health. These functions include production and secretion of bile, gluconeogenesis, biosynthesis of complex lipids, ureagenesis, and chemical detoxifications. In order to carry out their role in monosaccaride metabolism, liver cells have a high capacity complement of glucokinase, fructokinase C, and galactokinase 1. These enzymes are responsible for differential clearance of the common dietary hexoses from portal blood, initiating their conversion into glycogen for storage. This removal and storage is essential to glucose homeostasis and co-determines the postprandial blood sugar profile. One might be tempted to characterize liver as a hexose sensing organ/tissue because it contains sugar specific kinases that have suitable kinetic constants (S0.5 and Vmax values), are irreversible, and lack product inhibition. This meets a significant part of our definition of metabolic sensors. What distinguishes hepatocytes from cells of the endocrine-neuronal network is that expression of GCK in liver is absolutely insulin dependent and this expression is inhibited by glucagon. This is in contrast to constitutive expression of GCK in prototypical glucose sensing cells as, for example, in the islets of Langerhans and brain. The liver has a unique and crucial role in glucose homeostasis due to its large metabolic and storage capacities, but this role is regulated by insulin and glucagon, not glucose *per se*. The physiological importance of liver in glucose homeostasis is further attested by the presence of an additional insulin independent glucose sensing mechanism, inhibition of glycogen phosphorylase a (Hers, 1987; Matschinsky, 1990). Glucose at physiological concentrations facilitates enzymatic conversion of phosphorylase a to phosphorylase b and this inhibits glycogenolysis, an effect synergistic with glucose stimulation of glycogen synthesis.

The crucial role of GCK in control of hepatic fuel metabolism was recently demonstrated using mouse models in which hepatic insulin response was specifically eliminated by knockout of two Akt isoenzymes essential for insulin signaling. Akt double KO mice lack hepatic GCK, are hyperglycemic, hypolipidemic, and moderately ketotic but have very high serum insulin levels. Repairing the hepatic GCK deficiency by viral vector technology normalized the diabetic phenotype of double Akt knockout mouse (Titchenell et al., 2017). The role of GCK in liver is metabolic, i.e., it is important to postprandial removal of glucose from blood and maintaining hepatic glycogen levels. Its “regulatory” role is to activate metabolic pathways that store or degrade the G-6-P formed (glycogen synthesis vs glycolysis). Ferre et al. (1996) generated transgenic mice expressing the phosphoenolpyruvate carboxykinase/glucokinase (PEPCK/GCK) chimeric gene to study whether expression of GCK in the liver of diabetic mice might prevent diabetes induced metabolic alterations. In contrast to non-transgenic mice treated with streptozotocin, when mice with transgene were treated with streptozotocin their livers showed high levels of both GCK mRNA and activity. The increase in GCK was associated with an increase in intracellular levels of glucose-6-phosphate and glycogen as well as increase in pyruvate kinase activity and lactate production. In addition, in liver from streptozotocin-treated transgenic mice gluconeogenesis and ketogenesis were normalized. Thus, glycolysis was induced while gluconeogenesis and ketogenesis were blocked in diabetic mice expressing GCK. This was associated with normalization of blood glucose, ketone bodies, triglycerides, and free fatty acids even in the absence of insulin. Nissim et al. (2012) assessed the impact of activating GCK pharmacologically using isolated liver preparations of fed rats and perfusing them with 5 mM glucose and physiological concentrations of lactate, pyruvate and ammonia. They demonstrated that the allosteric GCK activator Piragliatin markedly enhanced every metabolic pathway downstream of glucose-6-phosphate that they studied. This included glycolysis, as indicated by a marked increase of lactate release and respiration associated with a clear increase in energy state, glycogen synthesis, lipogenesis and ureagenesis, apparently caused by increased levels of N-acetyl-glutamate, while inhibiting gluconeogenesis. These results attest to importance of GCK activity on hepatic intermediary metabolism. The study illustrates the biological significance of the GCK/GCKRP molecular complex as metabolic switch depicted in Figure 3.

## Establishing Causation: Glucose Sensitive Signaling Requires GCK

### The Kinetic and Biophysical Characteristics of Glucokinase Are Consistent With Its Glucosensor Role

Glucokinase functions as a monomer with a molecular weight of 55 K and the affinities (S0.5 and Km values) of the human enzyme for its two physiological substrates, d-glucose and Mg^2+^ATP, are about 8.0 and 0.4 mM, respectively (Meglasson et al., 1983; Meglasson and Matschinsky, 1986; Cárdenas, 2004; De Backer et al., 2016). The S0.5 for the other effective substrate d-mannose is about double that for d-glucose and stimulation of insulin release by this hexose has accordingly a right shifted dose response curve. Importantly, dependence of activity on glucose concentration is sigmoidal with a Hill number of 1.7 and this dependence is not significantly altered by products of the reaction. The inflection point in the substrate dependency curve (i.e., the steepest part of the sigmoidal curve) is about 4.0 mM, close to the glucose threshold for stimulation of insulin release. *In vivo*, the rate is determined by enzyme level and concentration of glucose, because [MgATP] remains near saturating levels and essentially constant. The cooperative (n = 1.7) dependence of activity on d-glucose concentration is mechanistically unique and consistent with the “mnemonic” or “ligand induced slow transition” models proposed by Cornish-Bowden and Cárdenas (1987) and by Neet (1980), respectively. These models [see also (Porter and Miller, 2012)] propose there are two different conformations of the enzyme, one with low and other high affinity for glucose, and conversion between them is induced by glucose. In the absence of glucose, GCK is in the low affinity conformation and binding to glucose induces the high affinity conformation. The rate of transition from low to high affinity forms is much slower than the catalytic cycle between substrate and product. These properties result in a sigmoidal dependence of the rate on glucose concentration. The dependence changes to hyperbolic when [MgATP] is below its Km. In liver, glucokinase regulatory protein (GCKRP) regulates GCK activity, but this is a special case as discussed earlier. Individuals carrying genetic mutations in GCK that result in altered kinetic constants present with symptoms consistent with predictions based on the measured changes (Osbak et al., 2009; Zelent et al., 2014).

### Genetic Alterations in GCK Directly Affect Glucose Homeostasis

Mutations of human GCK gene that alter its maximal activity and/or S0.5 for glucose result in disease states (Osbak et al., 2009). There are a wide variety of clinical manifestations depending on the mutation and whether one or both alleles are affected. More than 600 mutations have been discovered, beginning with reports that certain cases of MODY (maturity onset diabetes of the young) were linked to the GCK gene (Hattersley et al., 1992; Froguel et al., 1993). A vast majority of these mutations result in reduced enzyme capacity and cause a mild form of the disease (classified as MODY-2). As long as only one allele is affected blood glucose rises only slightly, by 1.0 mM or so, and these usually do not require therapy. If both alleles are affected, either by the same or different mutations, a severe form of diabetes, called permanent neonatal diabetes mellitus (PNDM), may result. This can be lethal if the babies are not treated with insulin as quickly as possible after birth. Mutations that increase maximal GCK activity or affinity for glucose cause hyperinsulinism (HI), the severity increasing with increase in enzyme activity or glucose affinity. In all cases of GCK linked HI currently known only one allele is involved. This suggests that, in humans, activation of both alleles causes fetal lethality whereas in mouse models homozygocity of strongly activating mutants is tolerated (Pino et al., 2007). The genetic characteristics of many of these mutations have been elucidated. The results are consistent with autosomal dominant inheritance mode of a gene encoding a protein essential to regulation of glucose homeostasis. Even minor changes in activity (e.g., ±20%) in one allele results in lowering or elevating blood sugar. The data from humans have been extensively studied and reproduced in mouse models. Rare mutations in the GCKRP gene are associated with mild forms of hyper-triglyceridemia and marginal lowering of blood glucose, consistent with this liver specific inhibitor (and liver glucokinase) playing an important, but secondary, role in glucose homeostasis in man. This is a view also supported by corresponding models in mice.

### Pharmacological Agents That Modulate GCK Activity Predictably Effect Blood Glucose Levels/Measured Cellular Activity

Activators/inhibitors of GCK have been used in many studies of the glucose sensitivity of individual cells, and have been particularly useful in studies of neurons. Typically, cellular responses to hyperglycemia can be mimicked by GCK activators and those of hypoglycemia by GCK inhibitors (Sweet et al., 1996; Grimsby et al., 2003; Matschinsky, 2009). This is considered strong evidence that GCK is responsible for the observed glucose dependences. Sweet et al. (1996) used d-mannoheptulose to study the effect of inhibiting glucose phosphorylation by GCK in cultured rat islets. They measured and mathematically modeled the effects of inhibition on the rate of glucose use and glucose oxidation as related to insulin release, providing compelling support for the GCK glucose sensor paradigm. Both basic science and clinical GCK studies have provided evidence that T2DM is associated with decreased GCK activity. This association has provided rationale for trying to find small molecules that activate GCK and thereby improve pharmacological treatment of T2DM (Grimsby et al., 2003; Matschinsky, 2009). High throughput screening based on glucokinase activity that is inhibited by acyl-CoA or GCKRP has identified a number of candidate molecules, i.e., molecules that activate GCK either indirectly through altered acyl-CoA binding or directly by binding to a hitherto unknown allosteric site of glucokinase. These GCK activators (GKAs) have been shown to lower blood sugar in various preclinical animal models and in human clinical trials. Several phase II studies have reported limited effectiveness and side effects that included hypoglycemia and hyperlipidemia (Matschinsky, 2013). These failures are perhaps not surprising considering the central role of GCK in an intricate regulatory network that includes a number of different target tissues. The dose response curves are biphasic and therapeutic range narrow. Furthermore, the target population of T2DM patients is usually heterogeneous and/or the disease progressed to the point where these drugs may not be indicated. Zhu et al. (2018), however, have reported very promising results in a phase II trial using Dorzagliatin to treat a non-obese, homogeneous, patient population of type 2 diabetics in China. Dorzagliatin is an allosteric GCK activator that activates both pancreatic and hepatic GCK. In addition, Ashton et al. (2014) has reported positive proof of concept for a novel drug that disrupts the inhibited GCK/GCKRP complex in liver. This drug prevents inactivation and nuclear sequestration of hepatic GCK when glucose is low and treatment of diabetic mice greatly improved blood glucose levels without observed negative side effects.

Study of human diseases linked to genetic alterations in GCK, together with pharmacological studies using GKAs, have led to discovery that GCK has a hitherto unrecognized allosteric activator site (see Figure 3). This site is in a region where there is a cluster of point mutations, mostly activating the enzyme, associated with human diseases and where GKA molecules bind. GKA binding is glucose dependent and the degree of dependence varies with chemical structure. GKA binding is facilitated by increasing glucose concentrations, potentially an important feature because it offers the possibility of designing GKAs with limited efficacy at low glucose. GCKRP also binds to the same region of GCK, but to a site distinct from GKA binding. GKA and GCKRP binding are mutually exclusive or at least operationally competitive.

## The Role of Individual Parts of the Signal Transfer Pathway, Downstream from GCK, in Glucose Homeostasis

It is clear that malfunction of any part of the signal transfer pathway that alters the relationship of GCK activity and/or the specific cellular response will result in disturbance of glucose homeostasis. Prominent among those parts for which malfunctions have been identified, are the monocarboxylate transporter (Otonkoski et al., 2007; Pullen et al., 2011) and the K^+^_ATP_ channel (Ashcroft et al., 1984; Ashcroft, 2005). In addition, alterations in metabolic pathways, other than GCK and glycolysis, that significantly influence the cellular energy state would be expected alter glucose homeostasis. The latter is exemplified by the consequences of genetic alterations in the activity of glutamate dehydrogenase (Fahien et al., 1988; Stanley et al., 1998; Wilson et al., 2018). The mechanisms by which malfunction in parts of the GCK signal transmission pathway, other than those in GCK itself, alter glucose homeostasis are not addressed in this paper. This does not suggest they are not important, just that they are not directly relevant to our hypothesis that GCK is the common glucose sensor for regulation of glucose homeostasis.

## On the Presence of Other Glucose Sensors of Physiological Significance, But not for Glucose Homeostasis

It is well recognized that taste receptors detect glucose and these are associated with the lining of the gut as well as the tongue, oral mucosa and olfactory system. Taste receptors have a broad specificity (McCaughey, 2008) and detect many molecules with different structures (fructose, maltose, glucose, sucrose, and artificial sweeteners) as sweet. Taste receptor molecular specificity is very different from that of the highly specific glucose sensing involved in glucose homeostasis. Taste receptors do not directly affect glucose homeostasis, but cells with sweet taste receptors do communicate with the brain and other organs involved in glucose homeostasis. Generally their influence is related to control nutrient intake from the environment and not glucose in the blood. If such non-specific receptors were to play a significant role in control of blood glucose this would introduce serious instability. It is not surprising that the gut has cells that use GCK for glucose sensing as well as cells with taste receptors.

Among the cells with specific sensitivity to glucose are many types of neurons that respond to changes in glucose concentration in the physiological range. Although most of these have GCK and appear to fit our hypotheses there are exceptions. Exceptions include the orexin/hypocretin neurons in the lateral hypothalamus (orexin neurons), GI neurons considered essential for normal cognitive arousal and feeding behavior (Sakurai et al., 1998; Yamanaka et al., 2003; González et al., 2008; Venner et al., 2011; Inutsuka and Yamanaka, 2013). Glucose sensitivity of orexin neurons has been studied in brain slices from mice in which these cells have been specifically targeted for expression of fluorescent proteins (Yamanaka et al., 2003). González et al. (2008) and Venner et al. (2011) reported that orexin neurons also respond to mannose, d-glucose, and 2-deoxyglucose but not galactose, L-glucose, α-methyl-d-glucoside, or fructose. Moreover, inhibition by glucose is not affected by inhibitors of GCK, indicating that in these particular neurons glucose sensing is not by GCK and metabolism of glucose is not required. It is not yet clear whether this is a receptor based glucose sensor, but the evidence is consistent with that conclusion. It is important to note that orexin neurons are primarily involved in regulating cognitive arousal, and there is no evidence that they play a significant role in glucose homeostasis. Thus, although glucose sensing by orexin neurons does not appear to involve GCK this does not represent an exception to our hypothesis.

## Is there Credible Evidence that Glucose Sensors Other than GCK are Important to Maintaining Glucose Homeostasis?

Although we feel the evidence makes it highly probable that our interpretation of the available data and the GCK glucose sensor hypothesis are correct, this is not a universal opinion. Recent reviews related to diabetes generally recognized GCK plays an important glucose induced insulin release by β-cells but some suggest the complexity of the system requires additional glucose sensitivity. Affourtit et al. (2018), for example, say “It is unlikely, however, that canonical glucose-stimulated insulin secretion is controlled by glucokinase alone, as hepatocytes express the same isozyme without bioenergetic glucose sensitivity.” Expecting the same response of energy metabolism to glucose in liver and β-cells because they both have GCK is clearly an “apples and oranges” error in logic. As discussed earlier, regulation of expression of GCK is different in β-cells and hepatocytes. In addition, hepatocytes, but not β-cells, express a specific GCK inhibitory protein. The metabolic roles of GCK in liver and pancreas are fundamentally different, one is in a large organ and responsible for major postprandial glucose uptake whereas the other is in endocrine cells and neurons that control release of potent regulatory hormones. The supporting metabolism (glycogen synthesis, glycolysis, fatty acid oxidation, etc.) is all different. Similarly, Nicholls (2016) states “However, this straightforward textbook scheme fails to account for many of the complexities surrounding physiological insulin secretion and its dysfunction in type 2 diabetes (T2D).” The nature of the failure(s) was not specified but contributions of mitochondrial bioenergetics were implied. Mitochondrial energy metabolism is an integral part of the metabolism downstream from GCK. Malfunction would affect glucose sensitivity, but that does not imply alteration of the primary glucose sensing process. No clear patterns or specific contributions to glucose sensing other than GCK have been found in the available reviews. It is reasonable to conclude that GCK is responsible for glucose sensing but there are mechanisms for “fine tuning” (or interfering with!) the response, particularly in the coupling of energy state to cell specific response. This type of ancillary input is essential to all biological sensor systems as they must be integrated into the rest of metabolism.

## Implications of Having a Single Glucose Sensor, GCK, in Understanding Glucose Homeostasis and Diabetes

**Figure 4:**
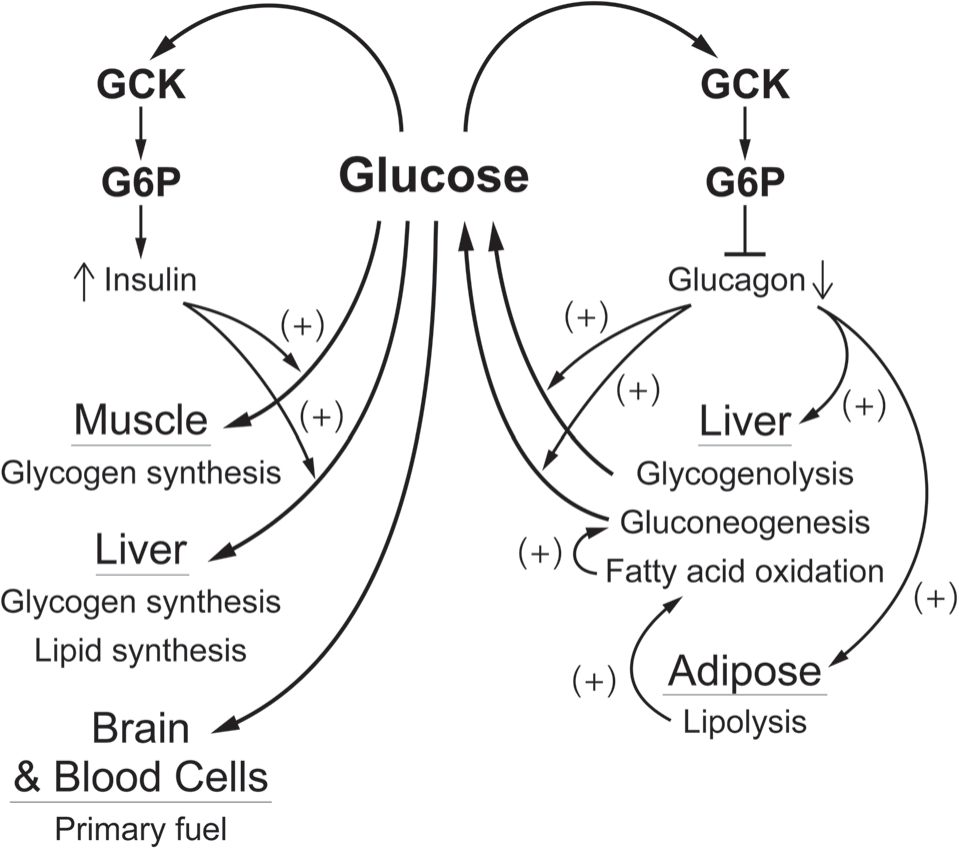
A schematic overview of how glucose regulates blood glucose by affecting α- and β-cells. The blood glucose level, through the activity of GCK which produces G6P as a “second messenger”, regulates release of glucagon from α-cells and of insulin from β-cells. Insulin is responsible for controlling consumption of glucose (removal from the blood), largely for storage as glycogen in muscle and liver. This is opposed by glucagon which is responsible for controlling production of glucose, mostly from liver glycogen and/or gluconeogenesis, and for activating fatty acid oxidation, primarily through production of fatty acids by adipose tissue. This “opposing forces” counter regulatory process is designed to achieve a balance (production equals consumption) at a blood glucose concentration near 5 mM.

As noted in the introduction, and schematically shown for the pancreas in Figure 4, regulation of glucose homeostasis based on a single glucose sensing system has important regulatory and medical consequences. Coupling of the signals for decreased blood glucose (insulin) and for increasing blood glucose (glucagon, epinephrine) to same glucose sensor provides a stable regulatory system with unique properties. Genetic alterations in the activity of GCK that increase or decrease in the affinity for glucose result in hypo- or hyperglycemia, which is typically, perhaps unintentionally, attributed to altered insulin secretion. It is, however, the regulatory set point for glucose homeostasis that is changed. The glucose concentration dependence of the counter regulatory hormones, notably glucagon and epinephrine, is also altered. This creates a problem when diabetes is due not to primary regulation but to other metabolic mechanisms, such as decreased insulin sensitivity of adipose and/or muscle tissue. Agents that lower blood glucose by activating GCK and increasing insulin secretion have undesirable effects, including suppressing counter regulatory hormone levels. Suppression of glucagon levels, for example, would be expected to potentiate hypoglycemia, a dreaded side effect of many antidiabetic agents, by suppressing glycogenolysis and gluconeogenesis, and hyperlipidemia by relieving inhibition of fatty acid synthesis. Thus, it is important to understand that GCK is the glucose sensor for regulation not only of release of insulin by β-cells but also for suppression of release of the counter regulatory hormones and glucose-dependent neural activity.

## Author Contributions

FM and DW contributed equally throughout preparation of this manuscript.

### Conflict of Interest Statement

The authors declare that the research was conducted in the absence of any commercial or financial relationships that could be construed as a potential conflict of interest.
